# Identification of a five-miRNA signature as a novel potential prognostic biomarker in patients with nasopharyngeal carcinoma

**DOI:** 10.1186/s41065-021-00214-9

**Published:** 2022-01-08

**Authors:** Bo Tu, Ling Ye, Qingsong Cao, Sisi Gong, Miaohua Jiang, Hui Li

**Affiliations:** 1 Department of Otorhinolaryngology and Head Neck Surgery, The first affiliated hospital of Jinan University, Guangzhou, 510630 Guangdong China; 2 Department of oncology, The first affiliated hospital of Jinan University, Guangzhou, 510630 Guangdong China

**Keywords:** Nasopharyngeal carcinoma, GEO, KEGG, miRNA, Prognostic

## Abstract

**Background:**

MicroRNAs (miRNAs) are involved in the prognosis of nasopharyngeal carcinoma (NPC). This study used clinical data and expression data of miRNAs to develop a prognostic survival signature for NPC patients to detect high-risk subject.

**Results:**

We identified 160 differentially expressed miRNAs using RNA-Seq data from the GEO database. Cox regression model consisting of hsa-miR-26a, hsa-let-7e, hsa-miR-647, hsa-miR-30e, and hsa-miR-93 was constructed by the least absolute contraction and selection operator (LASSO) in the training set. All the patients were classified into high-risk or low-risk groups by the optimal cutoff value of the 5-miRNA signature risk score, and the two risk groups demonstrated significant different survival. The 5-miRNA signature showed high predictive and prognostic accuracies. The results were further confirmed in validation and external validation set. Results from multivariate Cox regression analysis validated 5-miRNA signature as an independent prognostic factor. A total of 13 target genes were predicted to be the target genes of miRNA target genes. Both PPI analysis and KEGG analysis networks were closely related to tumor signaling pathways. The prognostic model of mRNAs constructed using data from the dataset GSE102349 had higher AUCs of the target genes and higher immune infiltration scores of the low-risk groups. The mRNA prognostic model also performed well on the independent immunotherapy dataset Imvigor210.

**Conclusions:**

This study constructed a novel 5-miRNA signature for prognostic prediction of the survival of NPC patients and may be useful for individualized treatment of NPC patients.

## Background

Nasopharyngeal carcinoma (NPC) occurs in the roof and lateral wall of the nasopharyngeal cavity. The common symptoms of NPC include nasal congestion, blood in the mucus, stuffiness in the ears, hearing loss, facial numbness, diplopia, and headache. It is associated with EBV infection, environment, genetics, smoking, and some other factors [1, 2]. NPC is particularly common in the Southern part of China and Southeast Asia, where it ranks first among head and neck malignant tumors in terms of morbidity and mortality [3, 4].

Despite sensitivity to radiotherapy and chemotherapy, the failure rate of nasopharyngeal cancer treatment remains high, with the main causes of failure being local recurrence and metastasis. Approximately 30–40% of patients with locally advanced NPC eventually develop distant metastases after radical treatment [5]. The median survival of patients with distant metastasis of NPC was only 19 ~ 21 months [6]. High risk of metastasis and recurrence of NPC will increase the complexity of and difficulty of treatment, and currently there is an urgent need to improve the treatment effect with effective biomarkers and treatment strategies. To achieve such a goal, it is very highly important to explore the biological and molecular mechanism of NPC and identify biomarkers related to the stratification of prognosis risk.

MicroRNAs (miRNAs) are a class of short, endogenous primed non-coding RNAs (18–25 nucleotides) that regulate gene expression through pairing with 3′-untranslated region (3′-UTR) bases to mediate translation inhibition or degradation of homologous mRNAs [7]. Extensive evidence suggested that miRNAs may act as tumor suppressor or oncogenes and play critical roles in the proliferation, invasion, apoptosis, differentiation and metabolism of tumor progression [8, 9]. In addition, about 50% of miRNAs were found to be located in “fragile loci” in the genome, which are most frequently amplified or lost in cancer.

It has been found that some miRNAs, including let-7a [10], miR-34 [11] and miR-93 [12], are associated with the progression of NPCs through regulating cell metastasis, growth, and apoptosis. 8-miRNA and 16-miRNA markers identified by Wen et al. can be used in diagnosing NPC, and these two are the first diagnostic markers of NPC screened from the whole blood sample so far. In particular, this study found that 16 miRNAs could effectively differentiate NPC from head and neck tumors [13]. Based on plasma EBVDNA and clinicopathological variables, a recent study established a nomogram as a more accurate predictor of NPC prognosis [14]. Ma and co-workers developed five miRNA signatures associated with NPC survival [15]. A recent study explored gene expression differences in patients with and without metastatic locally advanced NPC after treatment, and found a distant metastatic gene signature of 13 genes in locally advanced NPC, which has been considered as a reliable prognostic tool for recognizing distant metastasis of NPC [16]. Peter Shaw et al. also indicated prognostic significance of miRNAs as biomarkers in NPC patients [17]. These studies suggested that miRNAs could be expected to be effective prognostic markers and biological targets for NPC. The advantage of miRNA-based therapies is that miRNAs can simultaneously target multiple effector molecules in tumor cell differentiation and proliferation pathways. At present, these exploratory biomarker analyses are not sufficiently effective, thus, it is necessary to comprehensively explore the miRNA and regulatory mechanism related to NPC.

**Fig. 1:**
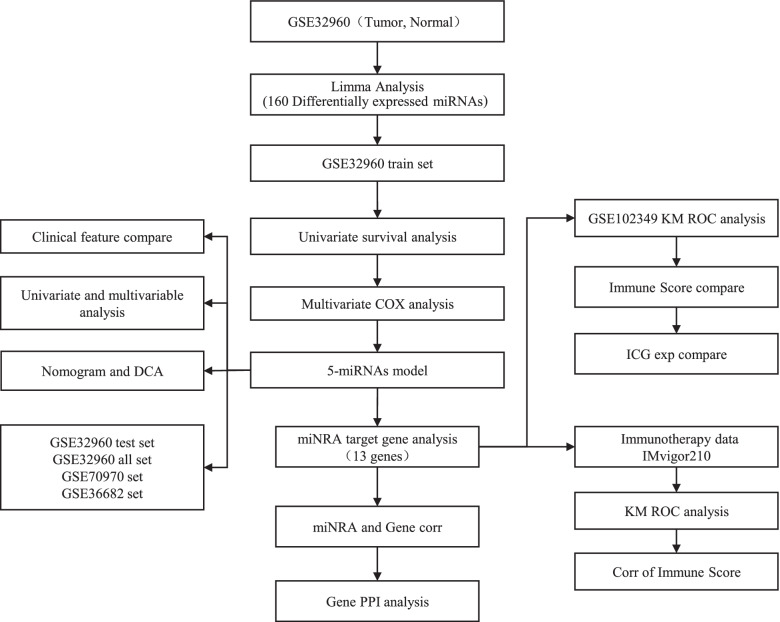
Work flow chart

This study applied bioinformatics to systematically analyze the microarray data of multiple gene expression profiles to screen the key genes, signal pathways and regulatory mechanisms in the occurrence and development of NPC (Fig. 1).

## Results

### Identification of differentially expressed miRNAs

**Fig. 2:**
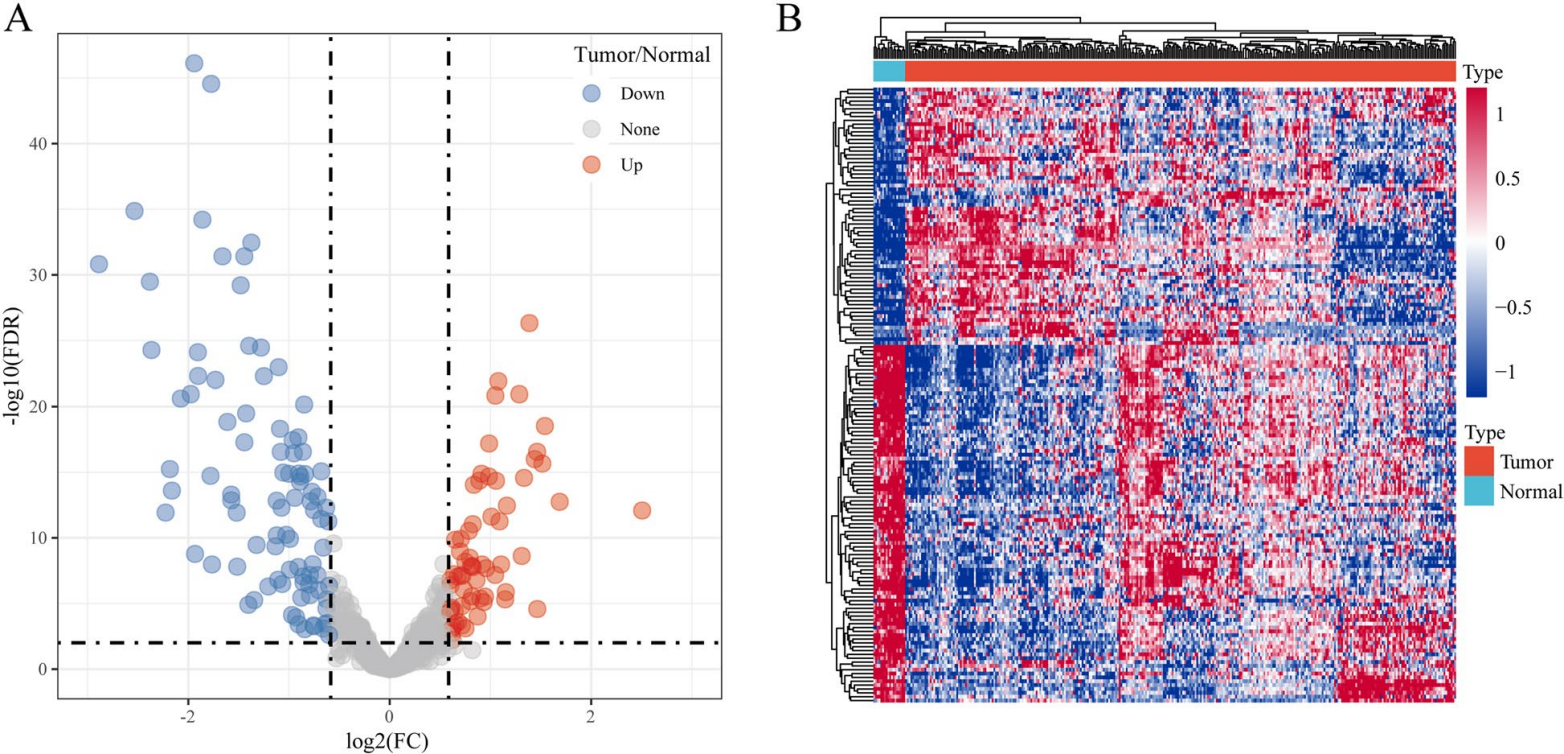
Identification of differentially expressed genes. A: Volcano map of differentially expressed genes between Tumor and Normal groups. B: Heat map of differentially expressed genes between Tumor and Normal groups

Between tumor and normal in the GSE32960 dataset, the limma package was used to calculate the differentially expressed miRNAs. A total of 160 differentially expressed miRNAs obtained included 67 up-regulated miRNAs and 93 down-regulated miRNAs (Fig. 2A, B). The univariate Cox proportional risk regression model was further conducted on the differentially expressed miRNAs using the R package survival coxph function, and we obtained a total of 11 prognostically related miRNAs here.

### Risk model construction and prognostic evaluation

**Fig. 3:**
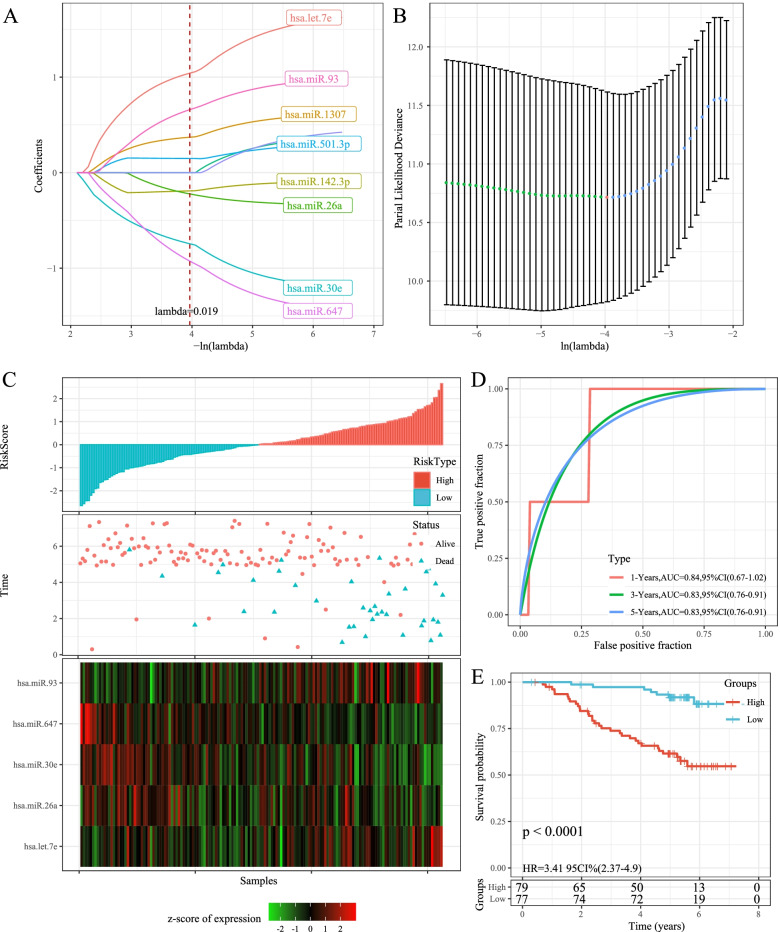
Risk model construction and prognostic evaluation. A: The change trajectory of each independent variable, the horizontal axis represents the log value of the independent variable lambda, and the vertical axis represents the coefficient of the independent variable; B: the confidence interval under each lambda. C: RiskScore, survival time and survival status and 5 miRNAs expression of each sample in GSE32960 training set. D: Classification ROC curve and AUC of 5-miRNA signature in GSE32960 training set. E: KM survival curve of 5-miRNA signature in the GSE32960 training set

For the 11 prognostic-related miRNAs, the R software package glmnet was used to perform lasso cox regression analysis. From the change trajectory of the independent variable, as the lambda gradually increased, the number of independent variable coefficients close to 0 also increased gradually (Fig. 3A). The model was constructed applying 10-fold cross-validation to determine the confidence interval of each lambda. The model was the optimal when lambda = 0. 019 (Fig. 3B). Hence, 8 miRNAs with lambda = 0.019 were chosen as the target genes. The stepAIC method in the MASS package further reduced 8 miRNAs to 5 miRNAs (hsa-miR-26a, hsa-let-7e, hsa-miR-647, hsa-miR-30e, and hsa-miR-93). The 5-miRNA signature formula was as follows:

$$\mathrm{RiskScore}=1.302\ast \left(\mathrm{hsa}-\mathrm{let}-7\mathrm{e}\right)-0.468\ast \left(\mathrm{hsa}-\mathrm{miR}-26\mathrm{a}\right)-1.108\ast \left(\mathrm{hsa}-\mathrm{miR}-30\mathrm{e}\right)-1.453\ast \left(\mathrm{hsa}-\mathrm{miR}-647\right)+0.88\ast \left(\mathrm{hsa}-\mathrm{miR}-93\right)$$

Risk score of each sample in the GSE32960 training set was calculated based on the expression level of genes. We divided the 406 patients into high- and low- risk groups with median risk scores as the cutoff value. OS of the low-risk group was obviously longer than the high-risk group. Heatmap of miRNA showed that miR-26a, hsa-miR-30e and hsa-miR-647 were protective factors, while hsa-let-7e and hsa-miR-93 were risk factors (Fig. 3C). The 1-year, 2-year and 5-year AUC for ROC curve of training data set was 0.84, 0.83 and 0.83, respectively (Fig. 3D). KM survival curve showed that the OS time in high-risk group had a shorter time than that in low-risk group (*p* < 0.0001, Fig. 3E).

### Robustness of risk models

**Fig. 4:**
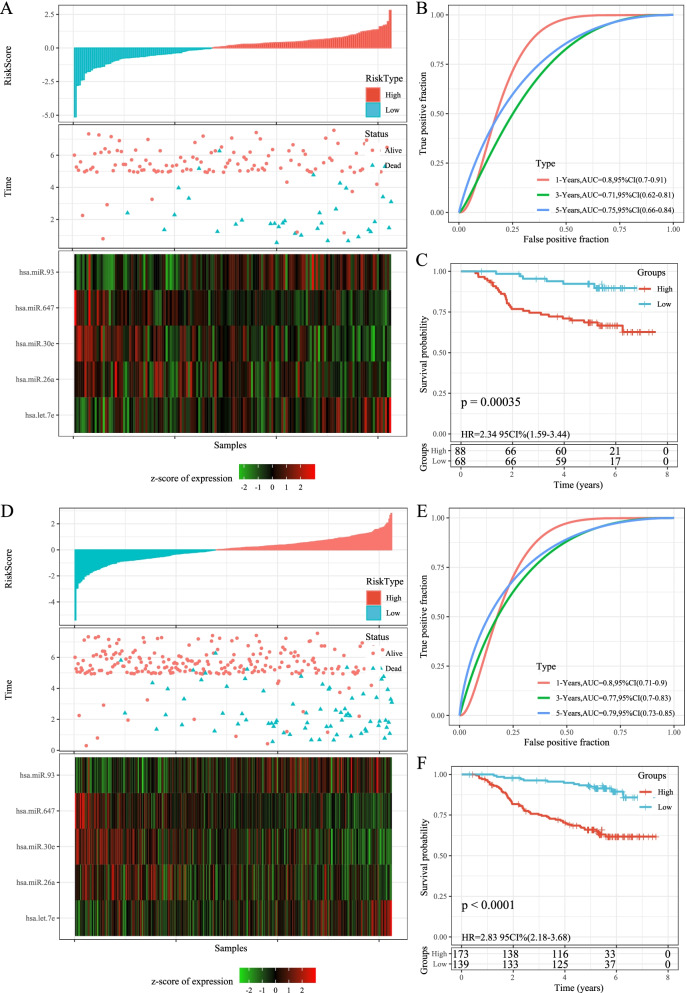
Robustness of risk models. A: RiskScore, survival time and survival status and 5 miRNAs expression of each sample in GSE32960 test dataset. B: classification ROC curve and AUC of 5-miRNA signature in GSE32960 test dataset. C: KM survival curve of 5-miRNA signature in the GSE32960 test dataset. D: RiskScore, survival time and survival status and 5 miRNAs expression of each sample in entire GSE32960 dataset. E: classification ROC curve and AUC of 5-miRNA signature in entire GSE32960 dataset. F: KM survival curve of 5-miRNA signature in the entire GSE32960 dataset

**Fig. 5:**
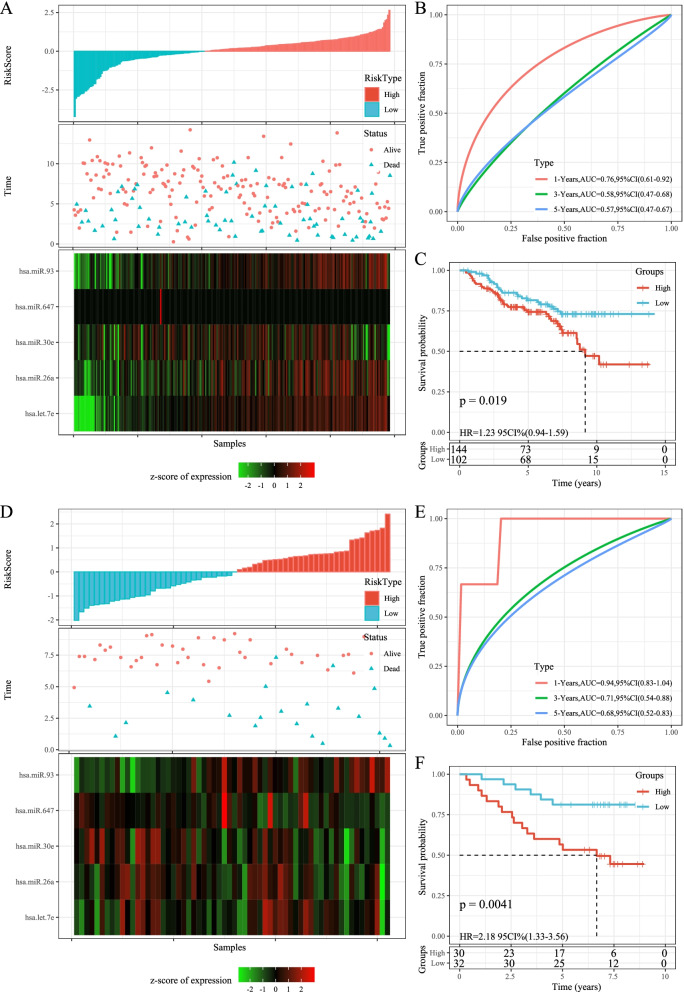
Robustness of risk models. A: RiskScore, survival time and survival status and 5 miRNAs expression of each sample in GSE70970 dataset. B: classification ROC curve and AUC of 5-miRNA signature in GSE70970 dataset. C: KM survival curve of 5-miRNA signature in the GSE70970 dataset. D: RiskScore, survival time and survival status and 5 miRNAs expression of each sample in GSE70970 dataset. E: classification ROC curve and AUC of 5-miRNA signature in GSE70970 dataset. F: KM survival curve of 5-miRNA signature in the GSE70970 dataset

To analyze the model robustness, we used the same model and coefficients in the validation set of GSE32960 and the full data set of GSE32960 as training set to calculate the risk score of each sample based on sample expression level, and the samples were classified into high-risk and low-risk groups based on the median value. Similarly, the OS of the low-risk group was obviously longer than that of the high-risk group. Heatmap of miRNA showed that miR-26a, hsa-miR-30e and hsa-miR-647 were protective factors, while hsa-let-7e and hsa-miR-93 were risk factors (Fig. 4A, D). The AUC for ROC curve of test dataset and entire GSE32960 dataset were all higher (Fig. 4B, E). KM survival curve also demonstrated that the OS time in high-risk group had a shorter time than that in low-risk group (Fig. 4C, F). The external datasets GSE70970 and GSE36682 were further used to verify the prognostic prediction ability of the gene signature. The OS of the low-risk group was obviously longer than that of the high-risk group in GSE70970 and GSE36682 dataset. Heatmap of miRNA showed that miR-26a, hsa-miR-30e and hsa-miR-647 were protective factors, while hsa-let-7e and hsa-miR-93 were risk factors (Fig. 5A, D). The AUC for ROC curve of GSE70970 and GSE36682 dataset were all higher (Fig. 5B, E). KM survival curve also showed that the OS time in high-risk group had a shorter time than that in low-risk group (Fig. 5C, F).

### Analysis of risk scores on clinical characteristics

**Fig. 6:**
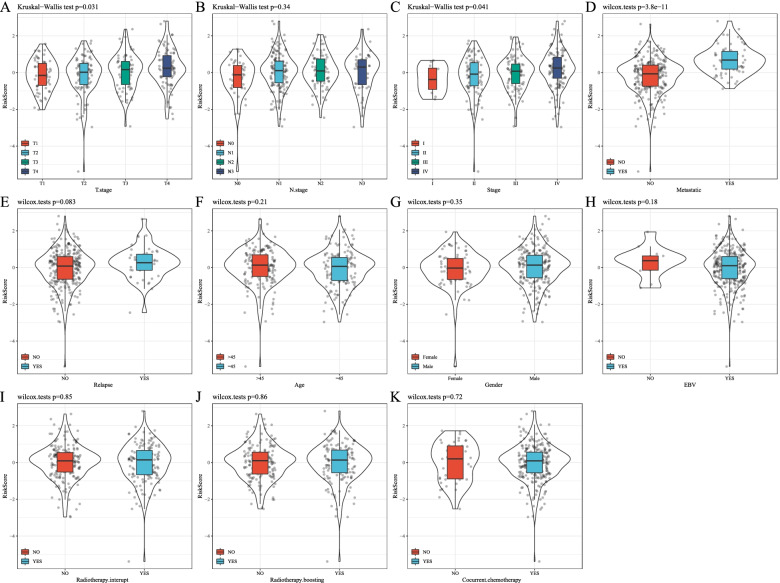
Analysis of Risk Scores on Clinical Characteristics. Comparison of the RiskScore among clinical feature groups in the GSE32960 dataset

Comparison of the distribution of RiskScore among clinical feature groups in the GSE32960 data set (Fig. 6) showed significant differences between T Stage, Stage, and the risk of developing metastasis (*p* < 0.05). More advanced stages such as T Stage and Stage were positively related to a higher risk score. For samples with metastasis risk, the risk score of the sample with metastasis was significantly higher than those without metastasis.

### Independence analysis of 5-miRNA signature

**Fig. 7:**
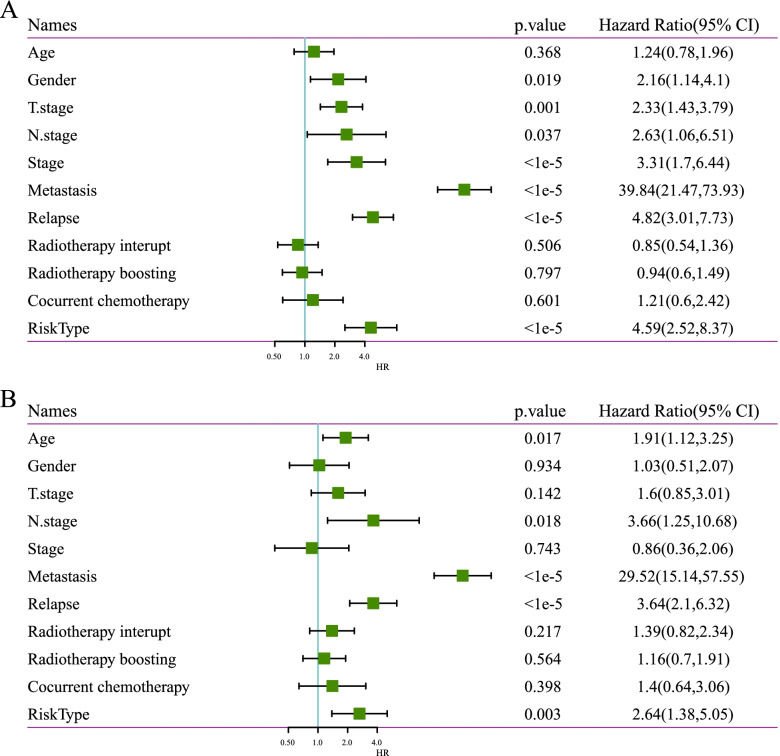
Independence analysis of 5-miRNA signature. A: Results of univariate analysis of clinical characteristics and RiskScore. B: Results of multivariate analysis of clinical characteristics and RiskScore

The relationship between prognostic indicators and clinicopathological characteristics was analyzed, including Age, Gender, T Stage, N Stage, Stage, Metastasis, Relapse, Radiotherapy interupt, Radiotherapy boosting, and Cocurrent chemotherapy. Univariate analyses demonstrated that RiskType, Stage, Metsastasisi, Replase were significant risk factors for poor outcome (Fig. 7A). Multivariate analysis indicated that a high riskscore and high Metsastasisi, Replase were independently associated with worse OS (Fig. 7B).

### Establishment of the nomogram and assessment of OS prediction

**Fig. 8:**
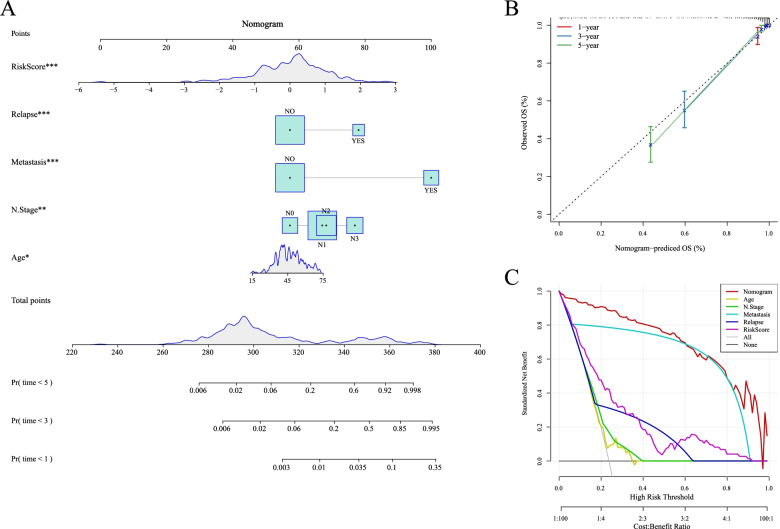
Establishment of the nomogram and assessment of OS prediction. A: A nomogram constructed by RiskScore and clinical features; B: A correction chart for survival rate of the nomogram; C: DCA curve

According to the results of univariate and multivariate Cox analysis, the 5-lncRNA signature was a reliable method to predict OS of patients with NPC. Then, according to the results from the multivariate Cox regression, we established a nomogram consisting of Age, N Stage, Metastasis, Relapse and RiskScore (Fig. 8A). The calibration plots verified the satisfactory predictive value between predictive values and observation values (Fig. 8B). The DCA curve also showed that RiskScore and Nomogram had a strong predictive effects (Fig. 8C).

### PPI analysis of miRNA target genes

**Fig. 9:**
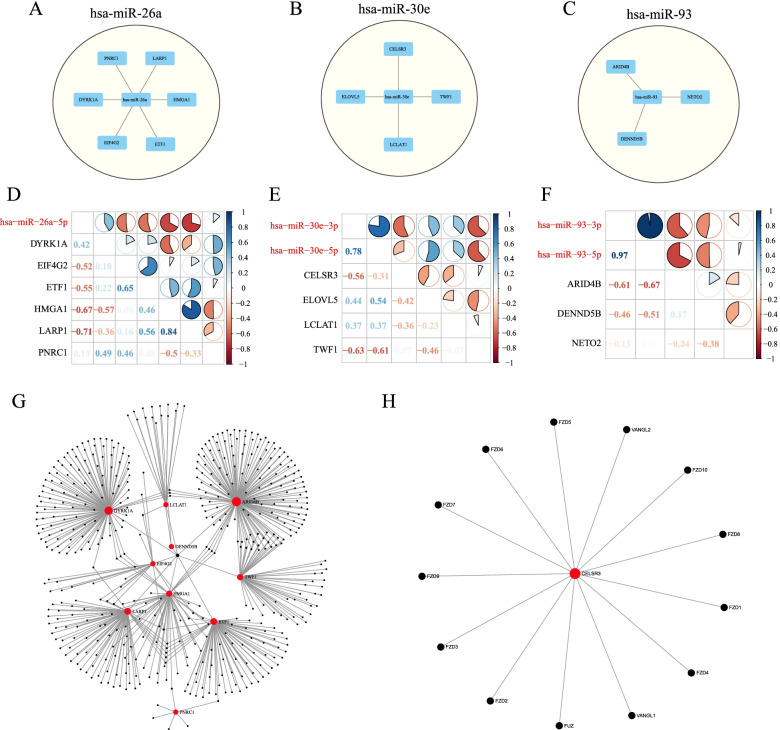
PPI analysis of miRNA target genes. A: Target gene prediction of miR-26a. B: Target gene prediction of miR-30e. C: Target gene prediction of miR-93. D: Correlation analysis between miR-26a and target genes. E: Correlation analysis between miR-30e and target genes. F: Correlation analysis between miR-93 and target genes. G: PPI network analysis of 13 target genes. H: PPI network analysis of 13 target genes

A total of 13 genes were predicted to be miRNA target genes, among them, 6 were the target genes of hsa-miR-26a, 4 were the target genes of hsa-miR-30e and 3 were the target genes of hsa-miR-93 (Fig. 9A-C). In the GSE118721 data set, Pearson correlation coefficients between miRNAs and corresponding target genes were calculated, and the results showed that most miRNAs were negatively correlated with target genes (Fig. 9D-F). PPI analysis was performed on the 13 target genes identified by miRNA analysis using NetworkAnalyst 3.0, and PPI correlation network analysis was performed using STRING. There were two networks, and KEGG pathway enrichment analysis was conducted using the related genes in the network. Subnet 1 annotated tumor-related pathways such as Wnt signaling pathway, Cell cycle, Prostate cancer, Pathways in cancer, Hepatitis C, Hepatitis B (Fig. 9G). Subnet 2 was annotated to Wnt signaling pathway, mTOR signaling pathway, Pathways in cancer, Proteoglycans in cancer and other tumor-related pathways (Fig. 9H).

### Prognostic effect and immune infiltration analysis of target gene model

**Fig. 10:**
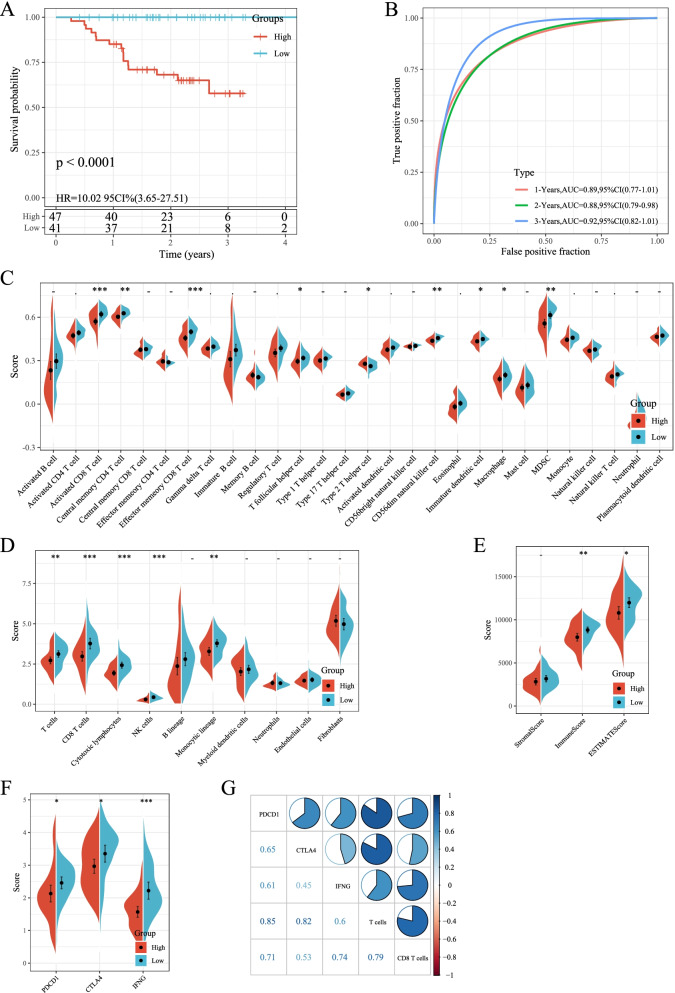
Prognostic effect and immune infiltration analysis of target gene model. A: KM survival curves for high and low risk groups in the target gene prognostic model in dataset GSE102349. B: ROC curves of the target gene prognostic model in dataset GSE102349. C: Comparison of ssGSEA immunization scores for high- and low-risk subgroups in the GSE102349 dataset. D: Comparison of MCPcounter immunization scores for high- and low-risk subgroups in the GSE102349 dataset. E: Comparison of ESTIMATE immunization scores for high- and low-risk subgroups in the GSE102349 dataset. F: Comparison of immunotherapy gene expression between high and low risk groups in GSE102349 data set. G: Correlation between immune checkpoint and immune score in GSE102349 dataset

Multivariate Cox analysis was performed on 13 target genes in the data set GSE102349 to obtain the risk coefficient of each gene. The samples were divided into high- and low-risk groups, and survival curve analysis and ROC curve analysis were performed. The results showed that the high- and low-risk groups of the data set GSE102349 had significant survival differences (Fig. 10A). The ROC curve of 1, 2, and 3 years had a high AUC value (above 0.88, Fig. 10B). The immune scores of the three software all demonstrated that the immune scores of the high-risk group were lower than those of the low-risk group (Fig. 10C, D, E). Furthermore, we compared the expression of PDCD1 (PD-L1), CTLA4 and IFNG (IFN-γ) genes in the high- and low-risk groups, and found that the expression of these three genes in the high-risk group was significantly lower than low-risk group (Fig. 10F). At the same time, correlation calculations between PDCD1 (PD-L1), CTLA4, and IFNG (IFN-γ) with T cells and CD8 T cells all showed a positive correlation (Fig. 10G). The above results indicated that low-risk grouped samples may have a better response to immunotherapy.

### Prediction of immunotherapy by risk modeling of target genes

**Fig. 11:**
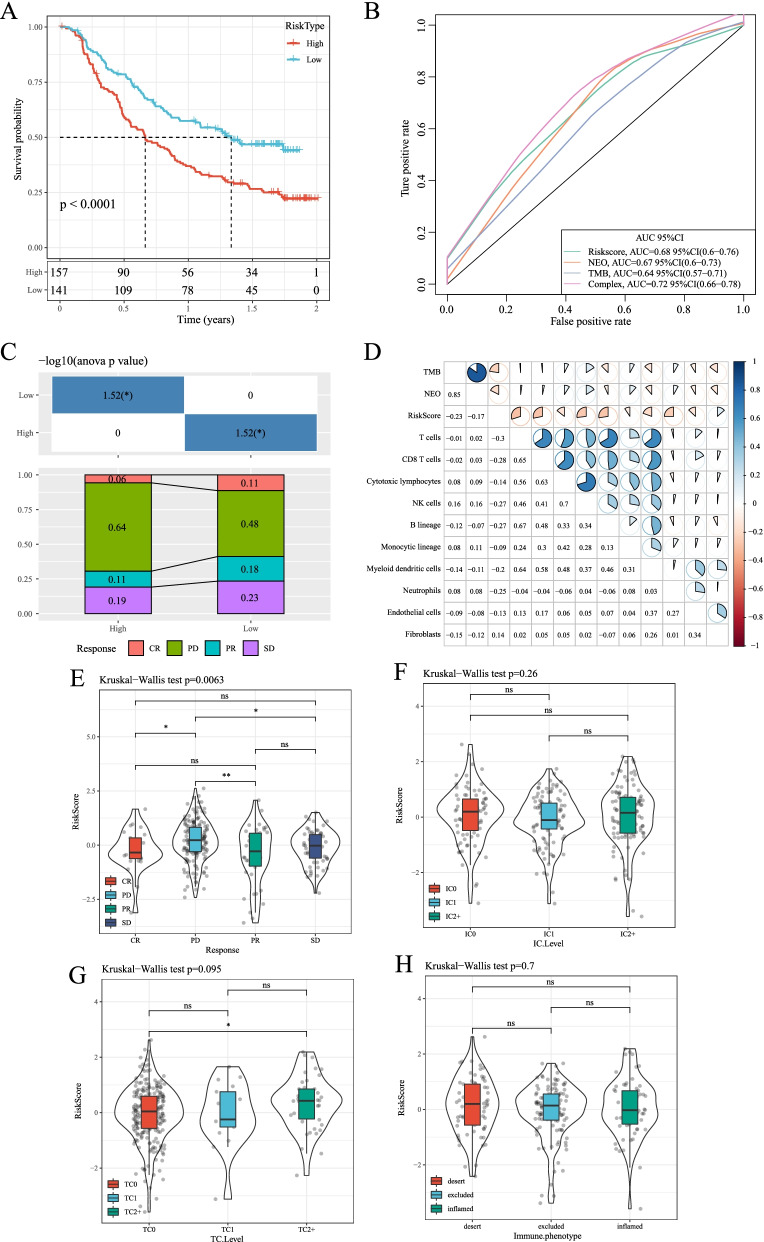
Prediction of immunotherapy by risk modeling of target genes. A: KM curve of target gene prognostic model in the Imvigor210 dataset. B: ROC curves of the target gene prognostic model in dataset Imvigor210. C: Comparison of immune cell scores between high-risk and low-risk groups in the Imvigor210 dataset. D: Comparison of immune cell scores between high-risk and low-risk groups in the Imvigor210 dataset. E: Comparison of StromalScore, ImmuneScore and ESTIMATEScore between high-risk and low-risk groups in the Imvigor210 dataset. F: Comparison of PDCD1, CTLA4 and IFNG expression between high-risk and low-risk groups in the Imvigor210 dataset. G: Correlation analysis between immune genes and immune cells in the Imvigor210 dataset

Currently, effective predictive markers for immunotherapy are limited. Identification of novel predictive markers is critical to further develop precision immunotherapy. We searched an immunotherapy dataset (Imvigor210), which stored expression data in human mUC samples with patients’ response to anti-PD-L1 immunotherapy. From Kaplan-Meier curves, it could be found that in mUC patients receiving immunotherapy, those with higher RiskScore values had poorer survival (Fig. 11A). The ROC curve showed that RiskScore had a higher AUC (Fig. 11B). Significant differences between immunotherapy responders and non-responders in high- and low-risk subgroups were detected, with a smaller proportion of patients in the high-risk subgroup showing response to immunotherapy (Fig. 11C). The immune cell scores of Imvigor210 samples were calculated using MCPcounter, and the correlation between RiskScore and TMB, NEO and immune cell scores was calculated. We found that RiskScore showed a negative correlation with TMB and NEO (Fig. 11D). At the same time, we compared the differences between RiskScore in different subgroups, and the data showed that RiskScore had significant differences in the response of effectiveness subgroup to immunotherapy (Fig. 11E), but RiskScore did not show significant differences between immune cell subgroup, tumor cells or immune phenotype (Fig. 11F-H).

## Discussion

Some of the key microRNAs identified in this study were consistent with other previous findings. Study showed miR-26a is downregulated in NPC and targets mRNAs, and that an increase in protein-encoding mRNAs may promote NPC invasion and metastasis [18, 19]. Therefore, downregulating miR-26a expression in NPC cells could contribute to NPC cell invasion and metastasis, leading to a poor overall survival. miR-93 was significantly upregulated in NPC cell lines and clinical specimens, and depletion of miR-93 inhibited NPC growth, invasion and migration in vitro and in vivo [12]. Li Jiang et al. reported that 3 small extracellular vesicles-derived miRNAs (miR-134-5p, miR-205-5p, and miR-409-3p) could potentially act as an alternative or complementary approach for diagnosing NPC [20]. Ma and co-workers identified five miRNAs (miR-142-3p, miR-29c, miR-26a, and miR-30e and miR-93) significantly associated with DFS as independent prognostic factors for NPC [15]. In contrast, this study again verified that miR-26a, miR-30e, and miR-93 could predict the prognosis of patients with NPC from multiple data sets, providing evidence for clinical diagnosis and treatment to find potential molecular markers and therapeutic targets. There is nearly no available research on the expression of hsa-miR-30e, hsa-miR-647 and hsa-let-7e in NPC, but they were the key microRNAs discovered in this study.

In this study, multiple databases were used to predict DEM target genes to improve the accuracy of predicting NPC prognosis. The intersection of DEM and differentially expressed genes of NPC were taken, a total of 13 intersection genes were found to be involved in the miRNA-mediated regulation of NPC. To understand the potential functional role of miRNA, target genes of miRNA were analyzed by PPI and KEGG functional annotation, and the results showed that DEG was mainly involved in Wnt signaling pathway, Cell cycle, mTOR signaling pathway, Pathways in cancer. Recently, Wnt/β-catenin as the key pathway for carcinogenesis has been reported to play a critical role in the induction and maintenance of EMT and LNM [21]. Interestingly, dysregulated signaling of the Wnt/β-catenin signaling pathway enhances the malignancy of many human cancers, including NPC [22]. The mTOR signaling pathway could promote cell proliferation and inhibiting apoptosis of NPC [23, 24]. The analysis of the signaling pathways involved in NPC-related intersection genes may provide new insights into the mechanisms of NPC cells.

## Conclusions

In summary, we successfully identified five miRNAs that may play a key role in the development of NPC metastasis using a comprehensive bioinformatics analysis. Hsa-miR-30e, HSA-miR-647 and HSA-let-7E, which have not been reported to be related to NPC, can be used as new potential markers of NPC. The regulatory role of relevant miRNAs in the mechanism of NPC occurrence and development and the detailed regulatory mechanism should be further studied. This study contributed to the promotion of individualized treatment of NPC in the future.

## Methods

### Data acquisition and processing

Clinical data and gene expression data of NPC were downloaded from the Gene Expression Omnibus (GEO, http://www.ncbi.nlm.nih.gov/geo/) database [25] including the GSE32960 [26], GSE70970 [27], GSE36682 [28], GSE118721, and GSE102349 [29] datasets. The GSE32960, GSE70970, and GSE36682 datasets were miRNA data containing clinical survival information; the GSE118721 dataset contained miRNA and mRNA data but without clinical survival information; and the GSE102349 dataset was mRNA data containing clinical survival information. The immunotherapy dataset used IMvigor210 [30] with expression data and clinical data.

For the GEO dataset, samples without clinical follow-up, survival time, or survival status were removed. Probes were converted to gene symbols. When a probe corresponded to multiple genes, the probe was removed, and the median expression value was taken from multiple gene symbols. For miRNA data, only human-associated miRNA expression data were retained. The sample information after data preprocessing is shown in Table 1. See Fig. 1 for the workflow diagram.

**Table 1:** Clinical information of the samples

Clinical Features	GSE32960(miRNA)	GSE36682(miRNA)	GSE70970(miRNA)	GSE102349(mRNA)
**Type**				
Normal	18			
Tumor	312	62	246	88
**OS**				
0	238	40	176	72
1	74	22	70	16
**Metastatic**				
NO	246			
YES	66			
**Relapse**				
NO	268			
YES	44			
**T Stage**				
T1	66			
T2	89			
T3	71			
T4	86			
**N Stage**				
N0	44			
N1	148			
N2	72			
N3	48			
**Stage**				
I	12			
II	86			
III	91			
IV	123			
**Gender**				
Male	233			
Female	79			
**Age**				
≤ 45	148			
>45	164			
**EBV**				
YES	299			
NO	13			
**Radiotherapy interupt**				
NO	178			
YES	134			
**Radiotherapy boosting**				
NO	163			
YES	149			
**Cocurrent chemotherapy**				
NO	44			
YES	268			

### Sample grouping

Firstly, 312 tumor samples from the GSE32960 data set were assigned into training set and validation set. All the samples were put back into random groups for 100 times to avoid random allocation bias that may affect the stability of subsequent modeling. Here, grouping sampling was conducted in accordance at the ratio of training set: validation set = 1:1. Here, follow-up time, sex, age distribution, and proportion of deceased patients were similar in the two groups. The two randomly assigned data sets were clustered and the number of dichotomous samples was similar to select the most appropriate training set and verification set. There are 156 samples in the final GSE32960 training set and 156 samples in the GSE32960 test set (Table 2). The training set and test set samples were subjected to Chi-square test, and no significant difference between groups was shown (*p* > 0.05).

**Table 2:** Sample Informations of GSE32960 Training Set and Validation Set

Clinical Features	GSE32960(train)	GSE36682(test)	P
**OS**			
0	117	121	0.6897
1	39	35	
**Metastatic**			
NO	122	124	0.8897
YES	34	32	
**Relapse**			
NO	132	136	0.6256
YES	24	20	
**T Stage**			
T1	30	36	0.7632
T2	48	41	
T3	36	35	
T4	42	44	
**N Stage**			
N0	23	21	0.7131
N1	73	75	
N2	39	33	
N3	21	27	
**Stage**			
I	5	7	0.8443
II	45	41	
III	47	44	
IV	59	64	
**Gender**			
Male	119	114	0.6025
Female	37	42	
**Age**			
≤ 45	78	70	0.2474
>45	78	86	
**EBV**			
YES	150	149	1
NO	6	7	
**Radiotherapy interupt**			
NO	90	88	0.9089
YES	66	68	
**Radiotherapy boosting**			
NO	85	78	0.4965
YES	71	78	
**Cocurrent chemotherapy**			
NO	26	18	0.2549
YES	130	138	

### Identification of differentially expressed genes

Limma package [31] were employed to calculate the differentially expressed miRNA between Tumor and Normal in the GSE32960 dataset under the condition of FDR < 0.05 and |log2FC| > 1.5.

### Cox risk analysis for univariate survival

Univariate Cox proportional risk regression models were constructed using the R package survival coxph function [32] for each differential miRNA as well as survival data in the GSE32960 training set. *p* < 0.05 was the threshold value.

### Model construction

To simplify the risk model, the genes obtained from univariate Cox analysis were further filtered by Lasso cox regression using the R package glmnet. In stepwise regression, AIC red pool information criterion, which considers the statistical fit of the model and parameters number, was used. The stepAIC method in the MASS package started with the most complex model and sequentially removed a variable to reduce the AIC, with a smaller value indicating a better model. Combining the expression of each prognosis-related gene, we developed an independent prognosis model. The *RiskScore* was calculated using the following formula:

$$\mathrm{RiskScore}=1.302\ast \left(\mathrm{hsa}-\mathrm{let}-7\mathrm{e}\right)-0.468\ast \left(\mathrm{hsa}-\mathrm{miR}-26\mathrm{a}\right)-1.108\ast \left(\mathrm{hsa}-\mathrm{miR}-30\mathrm{e}\right)-1.453\ast \left(\mathrm{hsa}-\mathrm{miR}-647\right)+0.88\ast \left(\mathrm{hsa}-\mathrm{miR}-93\right)$$

### Evaluation of the riskcore in GEO dataset

Each patient in the GSE32960 cohort, the GSE70970 dataset and GSE36682 dataset was assigned with a risk score using prognostic model. The median risk score as a cutoff was applied to classify NPC patient subjects into low-risk or high-risk group. We plotted survival curves using Kaplan-Meier (KM), and log-rank tests were conducted to evaluate survival differences in the two groups. Receiver operating characteristic curve (ROC) was established using “timeROC” software package [33], and we calculated area under the curve (AUC) value to analyze model sensitivity and specificity. In addition, a prognostic nomogram based on the Cox proportional hazards regression model was performed to visualize the correlation between individual predictors and survival in patients with NPC by using “rms” package of R software [34]. The performance of the prognostic line graph was assessed by C index and calibration curves.

Whether the model could be used as an independent prognostic factor was examined through including sex, age, T, M, and N, stage as independent variables. We also performed univariate cox regression analyses and multivariate cox regression analyses on changes of survival outcomes and survival time.

### Prediction of miRNA target genes

Target genes of the five prognostic-related miRNA genes were predicted using microT [35], miRanda [36], mircode [37], miRDB [38], miRmap [39], miRtarbase [40], PicTar [41], PITA, TargetMiner [42], and TargetScan [43]. Target genes predicted in all of the 10 databases were retained.

### Protein-protein interaction analysis

NetworkAnalyst 3.0 (http://www.networkanalyst.ca/) [44] is a web-based visual analysis platform for comprehensive interpretation of gene expression data at the level of system. We used NetworkAnalyst 3.0 to generate protein-protein interaction (PPI) network diagrams using experimentally validated data from STRING (version v11.0, https://string-db.org/) [45]. STRING (https://string-db) is a web-based visualization platform for comprehensive analysis and interpretation of system-level gene expression data. The database (.org/) is a searchable database of known interactions between proteins and predicted interactions between proteins. In PPI networks, biological systems are described based on proteins (e.g., nodes) and their relationships (physical/functional interactions) (e.g., edges), here each node represents a gene or protein and each edge represents an interaction between a pair of genes or proteins. Tools such as STRING databases and Cytoscape software are used for developing protein interaction networks for genes. Differentially expressed genes were subjected to KEGG pathway functional enrichment analysis in R software package WebGestaltR (V0.4.2) [46].

### Immunoassay

StromalScore, ImmuneScore, and ESTIMATEScore immune scores were examined using the R software package ESTIMATE [47]. 10 immune cell scores were analyzed by MCPcounter [48] and 28 immune cell scores were assessed using the ssGSEA method in the GSVA package [49].

### Statistical analysis

Benjamini & Hochberg was used for multiple inspection correction in the current study.

## Data Availability

The datasets generated and/or analyzed during the current study are available in the [GSE32960] repository [https://www.ncbi.nlm.nih.gov/geo/query/acc.cgi?acc=GSE32960], in [GSE70970] repository [https://www.ncbi.nlm.nih.gov/geo/query/acc.cgi], in [GSE36682] repository [https://www.ncbi.nlm.nih.gov/geo/query/acc.cgi], in [GSE118721] repository [https://www.ncbi.nlm.nih.gov/geo/query/acc.cgi?acc=GSE118721], and in [GSE102349] repository [https://www.ncbi.nlm.nih.gov/geo/query/acc.cgi].

## Abbreviations

miRNAs: MicroRNAs.
LASSO: Least absolute contraction and selection operator.
NPC: Nasopharyngeal carcinoma.
3′-UTR: 3′-untranslated region.
